# Supplementary material for: Atomic force microscope protocols for characterising the elastoviscoplastic biomechanical properties of corneocytes
Source: Commun Biol. 2025 Dec 4;8:1747. doi: 10.1038/s42003-025-09142-0 (PMC12678835; doi:10.1038/s42003-025-09142-0)
Supplement: Supplementary file 2 — Supplementary Material [file 42003_2025_9142_MOESM2_ESM.pdf]

Atomic force microscope protocols for characterising the elastoviscoplastic biomechanical properties of corneocytes.

Ana S. Évora, Zhihua Zhang, Simon A. Johnson, Zhibing Zhang, Michael J. Adams

School of Chemical Engineering, University of Birmingham, Birmingham, UK

Corresponding author: Michael J. Adams

Address: School of Chemical Engineering, University of Birmingham, UK

Email: m.j.adams@bham.ac.uk

Phone: +441214145297

Supplementary Information

Note: see the Appendix for the nomenclature used both here and in the paper.

S1 Methodology for calibration of AFM tip geometry using a reference elastomer

S1A. Sample

Sample: exposed-side of cast Sylgard 184 PDMS.

Elastic modulus, E^* = 3.79 ± 0.21 MPa (from micromanipulation measurements, See S5).

Poisson's ratio, ν = 0.5.

Environment: immersed in dilute surfactant to minimise adhesion forces.

S1B. Indentation test conditions and raw PDMS loading curves

AFM head displacement velocity = 500 nm/s.

AFM maximum force set point = 250 nN.

Probe force as a function of AFM head height.

S1C. Pre-analysis: zero force, contact point determination and compliance correction

- The cantilever stiffness (N/m) was obtained (from its resonance behaviour), as well as the cantilever deflection sensitivity (m/V) that are required for force calibration (N/V) using the JPK software. See S3 and Manuscript Methods.
- The indentation zero force for each loading curve is set by a baseline offset and tilt correction in the JPK software.
- The contact point of each PDMS loading curve is determined in Matlab from a linear fit of the initial 1–2 nN contact data (about 10 force/depth points spaced by 0.5 nm), on the assumption that the fit should extrapolate to F = 0 at h = 0. This allows the raw displacement data to be converted from the AFM head height to the probe indentation depth, after the probe height is compliance-corrected using the cantilever stiffness value. See Fig. S1.
- Only loading data for F > 1 nN (h > 5 nm, if the initial slope is ca. 0.2 N/m) are included in subsequent power law or polynomial fits (twice the noise level of 0.5 nN p-p), since points with F < 1 nN are assumed to be too susceptible to noise and local roughness.

S1D. Calibrated PDMS loading curves

Indentation force, F (nN), as a function of indentation depth (i.e. the total penetration depth), h (nm).

Number of data points = ca. 1000 per loading curve.

Number of curves = 64 in each of 3 zones.

Maximum, F_{max}= 250–253 nN (AFM set point = 250 nN).

Maximum indentation depth, h_{max} = 430–460 nm.

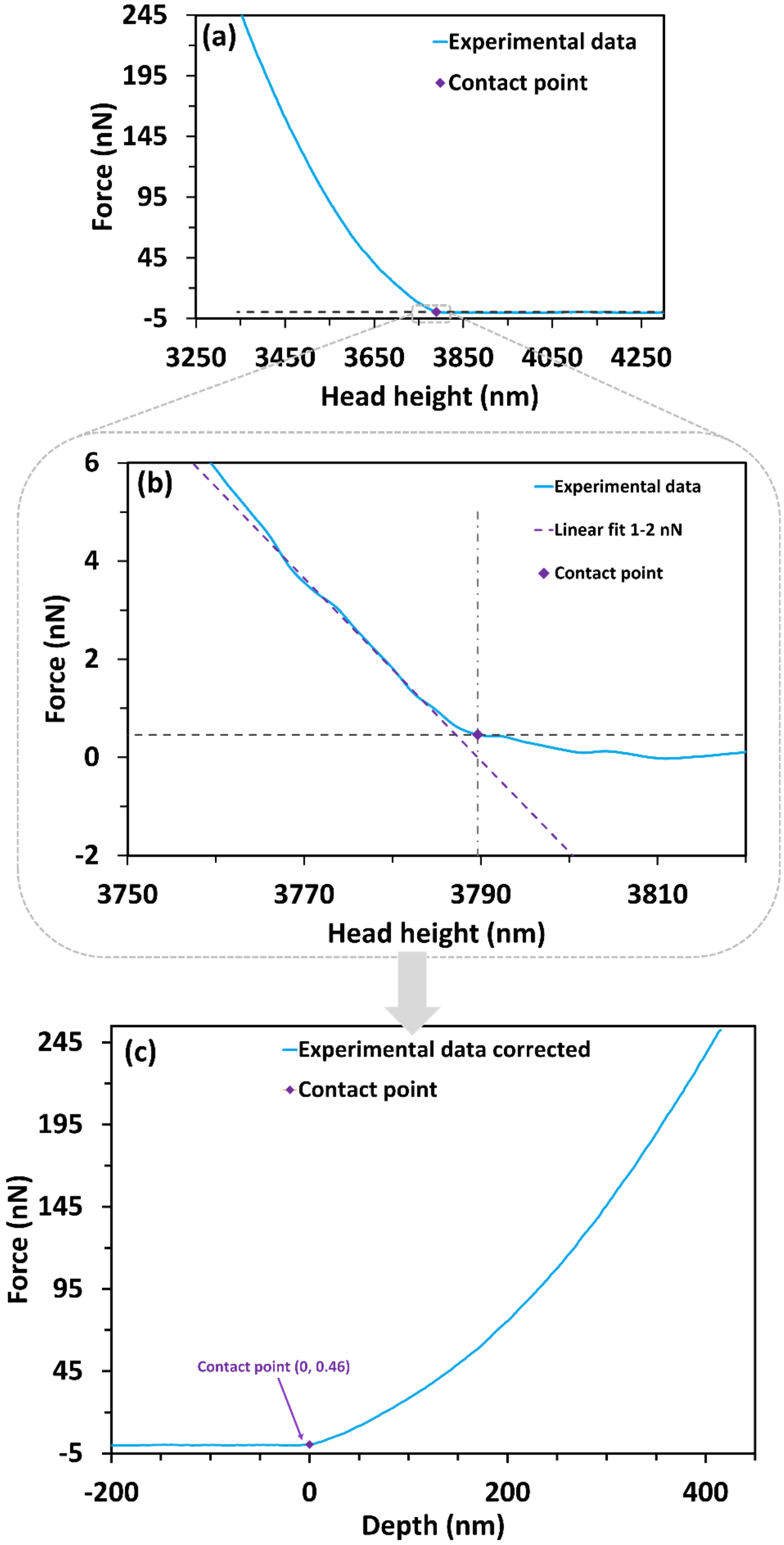

Fig. S1. (a) A single PDMS loading curve after baseline offset and tilt correction. **(b)** The contact point of each loading curve was determined from a linear fit of the initial 1–2 nN contact data, on the assumption that the fit should extrapolate to $F = 0$ at $h = 0$. **(c)** Compliance-corrected loading curve as a function of indentation depth, based on this estimate of the contact point which, in this example, is at 0 nm and 0.46 nN.

S1E. Power law fitting of a loading curve

$$F = bh^m \quad \text{S1}$$

$$S = \frac{dF}{dh} = mbh^{m-1} \quad \text{S2}$$

$$S = 2E^* a \quad \text{S3}$$

$$a = \frac{mbh^{m-1}}{2E^*} \quad \text{S4}$$

$$\frac{F}{S} = \frac{h}{m} \quad \text{S5}$$

$$m = \frac{Sh}{F} \quad \text{S6}$$

The experimental loading curves for PDMS of known elastic modulus fitted to Eq. S1 can be used to obtain the contact radius, a, as a function of indentation depth, h, using Eq. S4. However, for the current AFM probe, a single power law does not satisfactorily fit the data over the entire load range. Experimentally, the m value of the best-fit power law ranges from ca. m = 1.1 for fitting just the data close to contact, and towards ca. m = 1.7 for data at deep depths (h = 400 nm).

S1F. Two-term polynomial fitting of loading curve

$$F = b_1 h + b_2 h^2 \quad \text{S7}$$

$$S = \frac{dF}{dh} = b_1 + 2b_2 h \quad \text{S8}$$

$$S = 2E^* a \quad \text{S9}$$

$$a = \frac{b_1}{2E^*} + \frac{b_2 h}{E^*} \quad \text{S10}$$

A 2nd order polynomial with a zero intercept (Eq. S7) provides a more satisfactory fit over the entire range of a PDMS loading curve than a single power law (Eq. S1). Hence Eq. S10 implies that the probe geometry is better approximated by a truncated cone/pyramid rather than a single power law such as a paraboloid (*m* = 1.5).

S1G. Power law tip geometry, and loading curve analysis using a combination of power law equations and two term polynomial fits

$$h_c = ca^n \qquad \text{S11}$$

$$n = \frac{1}{m-1} \qquad \text{S12}$$

$$\kappa = \sqrt{\pi}\frac{\Gamma\left(\frac{n+2}{2}\right)}{\Gamma\left(\frac{n+1}{2}\right)} \qquad \text{S13}$$

$$h = \kappa h_c \qquad \text{S14}$$

$$a = \frac{mb\kappa^{m-1}h_c^{m-1}}{2E^*} \qquad \text{S15}$$

$$a = \frac{b_1}{2E^*} + \frac{b_2\kappa h_c}{E^*} \qquad \text{S16}$$

The tip geometry can also be described in power law form by Eq. S11. From the two fitted polynomial parameters, b_1 and b_2, Eq. S7 and Eq. S8 can be used to calculate predicted values of F and S as a function of indentation depth, h; namely $F(h)$ and $S(h)$. These predicted values of F and S can be used to calculate a number of parameters as a function of the indentation depth:

- $m(h)$: Eq. S6
- $b(h)$: Eq. S1
- $a(h)$: Eq. S4
- $n(h)$: Eq. S12
- $\kappa(h)$: Eq. S13
- $h_c(h)$: Eq. S14

These allow $a(h)$ to be expressed as $a(h_c)$ in Eq. S16, where the semi-included angle, α, of the equivalent cone is given by $\tan^{-1}\alpha = b_2\kappa/E^*$ and the radius of the truncation is given by $a(h_c = 0) = b_1/2E^*$. The tip contact radius function, $a(h_c)$, is a description of the tip geometry and can be expressed, equivalently, by Eq. S15 where b, m and κ vary continuously as a function of h. Essentially, for each point on a predicted loading curve generated from a polynomial fit, $F(h)$, there is a unique power law that gives the same predicted force and slope (contact stiffness) at the same indentation depth. Similarly, for each point on the tip radius function, $a(h_c)$ there is a unique power law that gives the same predicted contact radius and slope at the same contact depth, h_c.

For corneocytes, the Oliver & Pharr analysis [1] for an elastoplastic contact is used to obtain the value of h_c associated with h_{max} by measuring the initial unloading stiffness S_0. Eq. S16 can then be used to obtain *a* from h_c. The elastic modulus then follows from Eq. S9.

S1H. Power law tip geometry, and loading curve analysis using a combination of six-term polynomial smoothing and power law equations

$$F = b_1 h + b_2 h^2 + b_3 h^3 + b_4 h^4 + b_5 h^5 + b_6 h^6 \quad \text{S17}$$

$$S = \frac{dF}{dh} = b_1 + 2b_2 h + 3b_3 h^2 + 4b_4 h^3 + 5b_5 h^4 + 6b_6 h^5 \quad \text{S18}$$

$$S = 2E^* a \quad \text{S19}$$

$$a = \frac{b_1}{2E^*} + \frac{b_2 h}{E^*} + \frac{3b_3 h^2}{2E^*} + \frac{2b_4 h^3}{E^*} + \frac{5b_5 h^4}{2E^*} + \frac{3b_6 h^5}{E^*} \quad \text{S20}$$

$$a = d_0 + d_1 h_c + d_2 h_c^2 + d_3 h_c^3 + d_4 h_c^4 + d_5 h_c^5 + d_6 h_c^6 \quad \text{S21}$$

A significantly better fit to each loading curve results from using the 6th order polynomial with a zero intercept as given by Eq. S17. In particular, this reduces small, but significant, percentage systematic deviations between experimental loading curves and those predicted using Eq. S7 in the region 20 nm < h < 200 nm. These are attributed to deviations of the actual tip shape from a perfect flat-ended truncated cone. Any residual deviations are random, and a 6-term polynomial reduces each *ca.* 1000 point experimental loading curve to a set of 6 coefficients. This procedure acts as a smoothing function for the entire loading curve without residual tip-shape related systematic deviations.

By analogy with the procedures described above in S1G, the following steps are taken to numerically generate the tip function from a single AFM loading curve. All except the first step were implemented in Matlab.

A. Force zero set by tilt and offset correction in JPK software (see S1C).
B. Contact point set by linear back extrapolation of first 1–2 nN of data (see S1C).
C. 6th order polynomial with zero intercept fit from 1 nN and upwards of entire loading curve.
D. Create ca. 450-point arrays from h = 1 nm to $h = h_{max}$ in 1 nm steps using 6 fitted coefficients of $F(h)$ using Eq. S17.
E. $S(h)$: Eq. S18

F. $m(h)$: Eq. S6

G. $b(h)$: Eq. S1

H. $a(h)$: Eq. S4

I. $n(h)$: Eq. S12

J. $\kappa(h)$: Eq. S13

K. $h_c(h)$: Eq. S14

L. $a(h_c)$: Eq. S15. This tip contact radius function is valid for h = 5 nm to h_{max}, since it is the range in which the polynomial fit to the loading curve is confined.

M. $c(h_c)$: Eq. S11

N. 6th order polynomial fit with fitted intercept (Eq. S21) of $a(h_c)$ from h_c = 10 to 200 nm, which includes the complete range of values obtained from the Oliver & Pharr analysis of corneocytes.

S1I. Tip contact radius function and corneocyte indentation

Multiple loading curves with the AFM probe for the PDMS specimen are averaged after performing Step D on them all, and this mean loading curve $F(h)$ is processed through the subsequent steps to produce a mean tip contact radius function array for fitting in Step N. The resulting 7 coefficients from an example fit to Eq. S21 are shown in Fig. S2. The parameter arrays from intermediate steps can be used to calculate the mean and standard deviations, for example, at specific h or h_c values as shown in Table S1. These coefficients can then be used to calculate *a* from h_c obtained from corneocyte unloading curves using this AFM tip and the well-established Oliver & Pharr elastoplastic analysis procedure (see Fig. 4a).

S1J. Assumptions:

- Eq. S13 can be used in Eq. S14, even when the actual indenter tip shape has n varying with h_c.
- The Young's modulus of cast Sylgard 184 is independent of depth (See S5).

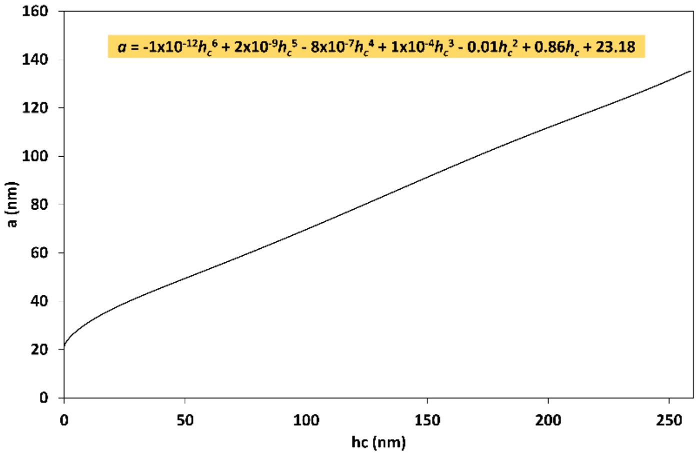

Fig. S2. The tip contact radius function for zone 3 of PDMS (average of 64 loading curves), which was obtained from a fit of $a(h_c)$ values from h_c = 10 to 200 nm.

Table S1. The parameters of the indenter geometry obtained from an elastic analysis of PDMS. The power law load index, m, was calculated at different indentation depths based on polynomial fitting of multiple loading curves. The derived parameters c and n define the geometry of the AFM tip at different contact depths, h_c.

h_c (nm)	m	n	c (μm^{1-n})
**5**	1.150 ± 0.025	7.55 ± 0.20	(7.90 ± 3.90) x 10^{-11}
**10**	1.184 ± 0.004	5.41 ± 0.10	(7.80 ± 3.11) x 10^{-8}
**15**	1.217 ± 0.005	4.62 ± 0.11	(1.26 ± 0.40) x 10^{-6}
**20**	1.240 ± 0.004	4.61 ± 0.08	(5.90 ± 1.31) x 10^{-6}
**30**	1.278 ± 0.003	3.59 ± 0.04	(4.55 ± 0.63) x 10^{-5}
**40**	1.311 ± 0.003	3.21 ± 0.03	(1.85 ± 0.21) x 10^{-4}
**50**	1.344 ± 0.002	2.90 ± 0.02	(5.89 ± 0.50) x 10^{-4}
**60**	1.380 ± 0.002	2.63 ± 0.01	(1.65 ± 0.01) x 10^{-3}
**70**	1.414 ± 0.002	2.42 ± 0.01	(3.85 ± 0.20) x 10^{-3}
**80**	1.450 ± 0.002	2.22 ± 0.01	(8.26 ± 0.20) x 10^{-3}
**90**	1.484 ± 0.003	2.07 ± 0.01	(1.56 ± 0.05) x 10^{-2}
**100**	1.518 ± 0.004	1.93 ± 0.02	(2.70 + 0.11) x 10^{-2}
**110**	1.550 ± 0.005	1.82 ± 0.02	(4.31 ± 0.21) x 10^{-2}
**120**	1.580 ± 0.006	1.73 ± 0.02	(6.38 ± 0.32) x 10^{-2}
**130**	1.605 ± 0.006	1.65 ± 0.02	(8.63 ± 0.42) x 10^{-2}
**140**	1.628 ± 0.006	1.59 ± 0.01	0.112 ± 0.005
**150**	1.648 ± 0.005	1.54 ± 0.01	0.139 ± 0.005
**160**	1.663 ± 0.005	1.51 ± 0.01	0.162 ± 0.005
**170**	1.674 ± 0.004	1.48 ± 0.01	0.180 ± 0.003
**180**	1.680 ± 0.003	1.47 ± 0.01	0.192 ± 0.003
**190**	1.683 ± 0.003	1.46 ± 0.01	0.197 ± 0.005
**200**	1.682 ± 0.003	1.47 ± 0.01	0.196 ± 0.007
**210**	1.681 ± 0.004	1.47 ± 0.01	0.19 ± 0.01
**220**	1.682 ± 0.006	1.47 ± 0.01	0.20 ± 0.01
**230**	1.687 ± 0.009	1.46 ± 0.02	0.20 ± 0.02
**240**	1.695 ± 0.012	1.44 ± 0.03	0.22 ± 0.02
**250**	1.700 ± 0.014	1.41 ± 0.04	0.26 ± 0.04

S2 Methodology flow chart for corneocyte extraction and analysis

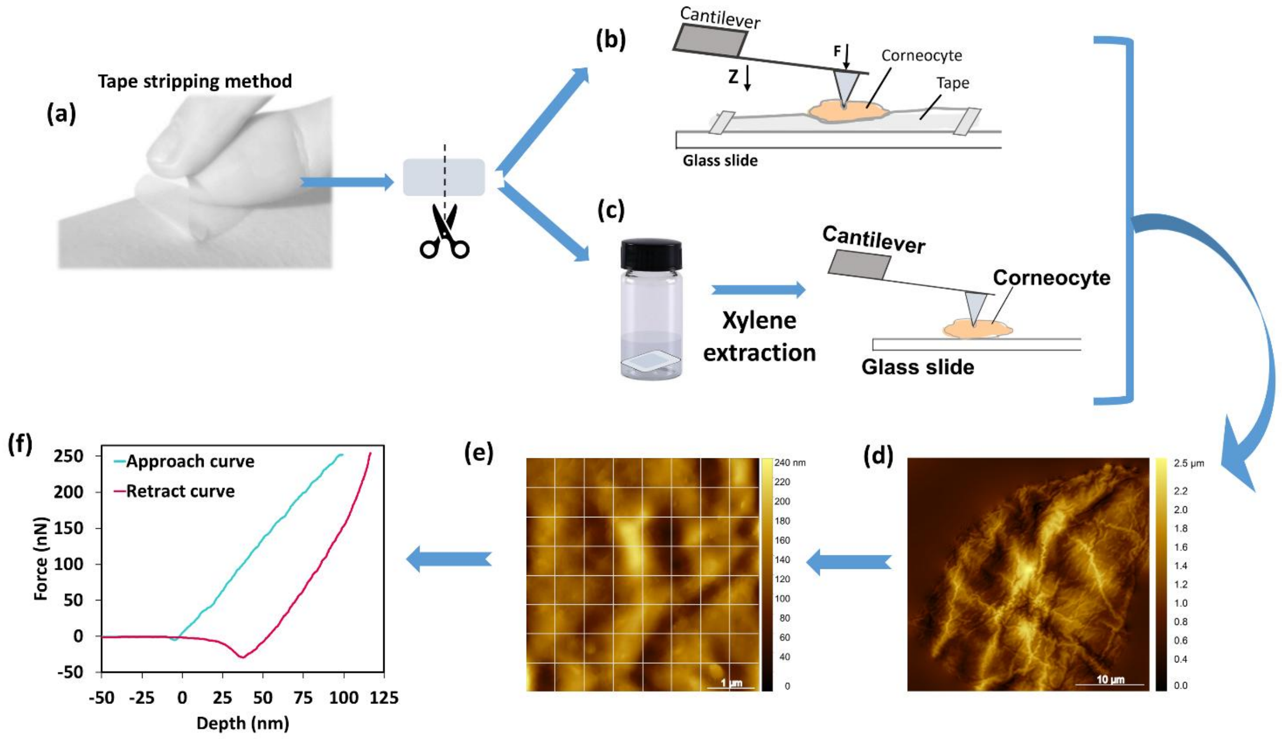

Fig. S3. The workflow for the collection and analysis of corneocytes using AFM: **(a)** the tape stripping method was used to collect cells from the volar forearm of 3 healthy subjects using regular Sellotape and (**b**) half of the tapes were used directly for AFM measurements. **(c)** The remaining half of the tapes were pressed on glass slides and immersed in xylene overnight. This removed the tape and adhesion glue, transferring the corneocytes onto the slides. **(d)** Topographical images of single corneocytes were obtained using AFM tapping mode. **(e)** Zoom-in image (5 × 5 µm) showing the 64 force curves grid used for the force spectroscopy experiments. **(f)** Example of loading and unloading curves obtained for a typical corneocyte attached to tape.

S3 AFM sensitivity calibration

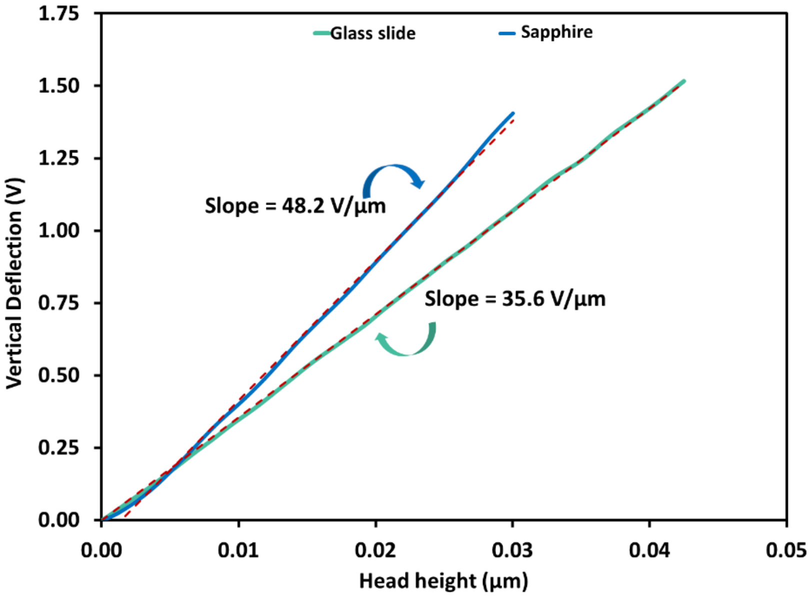

Fig. S4. The AFM cantilever deflection sensitivity (nm/V) was calibrated after each set of experiments by pressing the tip onto a relatively rigid material, on the basis that there should be relatively small amounts of indentation accompanying the deflection. Representative examples of the calculated sensitivity values were 28.1 nm/V (slope = 35.6 V/µm) for a glass microscope slide and 20.7 nm/V (slope = 48.2 V/µm) for a sapphire sample. Sapphire was chosen for sensitivity calibration due to its greater stiffness compared to the glass.

S4 Attenuated Total Reflection Fourier Transform Infrared (ATR-FTIR) spectroscopy

To explore any effects of xylene immersion, corneocytes were analysed using ATR-FTIR spectroscopy with a Nicolet 860 Fourier transform infrared microscope with OMNIC software on the computer interfaced to the spectrometer (Thermo Fisher Scientific UK Ltd., Loughborough, Leicestershire, LE11 5RG, UK). The spectra of corneocytes were obtained on tape, and after xylene extraction and re-adherence to the tape. In addition, spectra of the tape were acquired. Each spectrum consisted of 64 scans recorded in the wave-number range 600–4000 cm^{-1} and with a resolution of 4 cm^{-1}. Peak identification was done by consulting the literature [2, 3]. Fourier self-deconvolution (FSD) of the infrared spectra covering the Amide I region (1595−1705 cm^{-1}) was conducted using OMNIC software (Thermo Scientific, USA). Deconvolution was automatically performed using the Gaussian/Lorentzian line shape with a noise reduction factor of 0.3. The peak band assignment was based on literature [4].

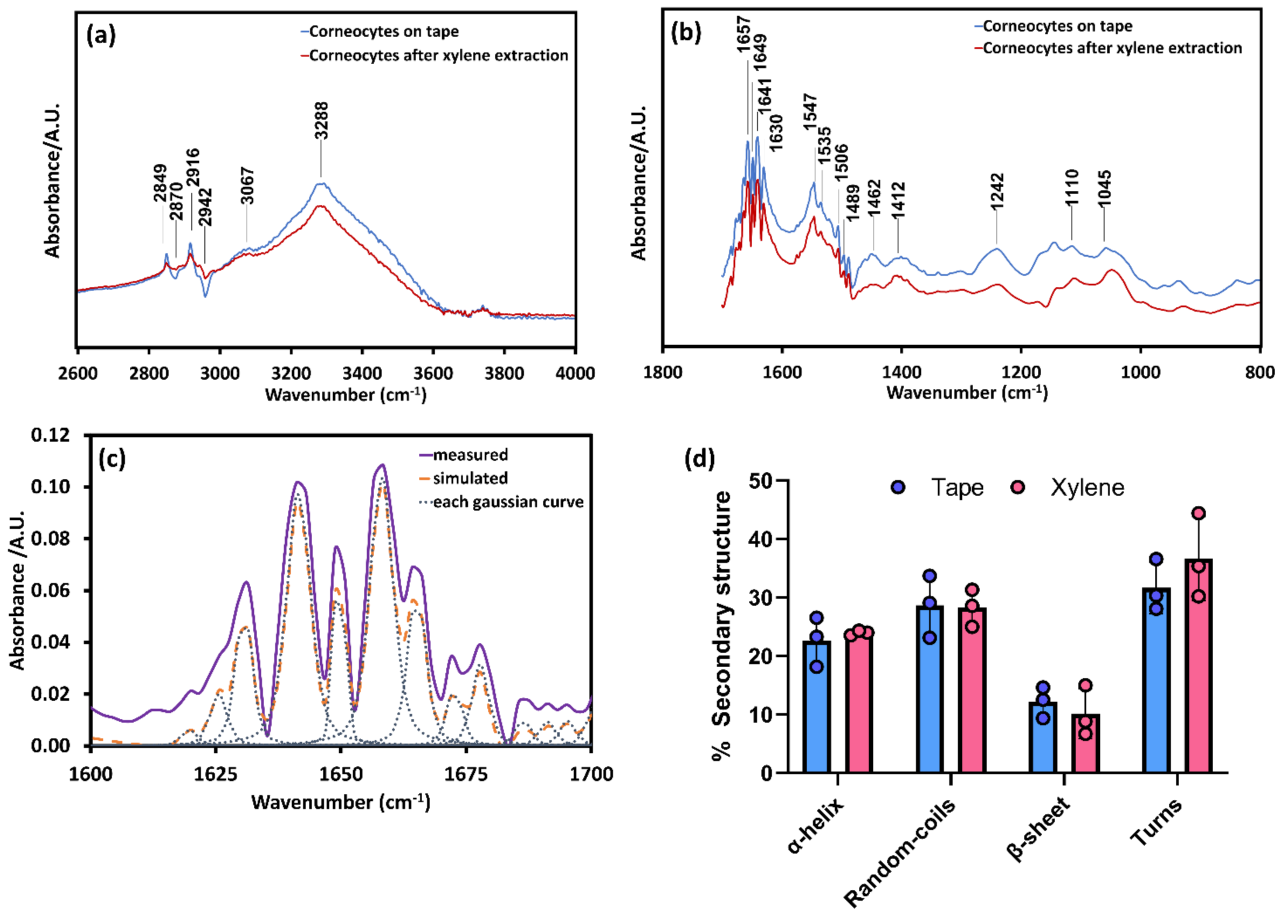

Fig. S5. (a) and (b) attenuated total reflection Fourier transform infrared (ATR-FTIR) absorbance spectra of corneocytes (on tape) before and after xylene extraction in the spectral region of **(a)** 2600–4000 cm^{-1} and **(b)** 800–1700 cm^{-1}. **(c)** a typical example of multiple Gauss curve-fitting to corneocyte ATR-FTIR spectrum for the Amide I band (1595–1705 cm^{-1}) spectrum. **(d)** secondary structures content based on Amide I band deconvolution for corneocytes on tape and after xylene extraction. Data represents n = 3 biological replicates. 2-way ANOVA was performed followed by Bonferroni's test with no statistical significance found between secondary structures. Error bars represent ± 1SD.

Typical ATR-FTIR spectra for corneocytes before and after xylene extraction are shown in Figs S5a and S5b and the main peaks are identified and assigned in Table S2. There were not any apparent differences observed in the spectra for the range 700–1700 cm^{-1} and 2600–3800 cm^{-1}. The more prominent bands were consistent with the work of Garidel [2] for corneocytes extracted from SC sheet, by removing the lipid fraction with chloroform. Furthermore, the band found at 1410.5/1412 cm^{-1} was attributed to amino acids belonging to the Natural Moisturising Factor (NMF), as described by Takada et al. [4]. Corneocytes are mainly composed of fibrous proteins like keratins and filaggrin and thus, to account for the secondary structure of the proteins, a deconvolution of the Amide I band was done to evaluate any effect of the xylene extraction. In Fig. S5c, a typical example of multiple Gauss curve-fitting is shown. Xylene did not seem to affect the secondary structure of proteins since there was not any statistical differences found before and after tape extraction (Fig. S5d).

Table S2. Assignments of the most prominent infrared absorption bands of corneocyte spectra on tape and after xylene extraction.

Tape		Xylene extraction	
**Frequency (cm^{-1})**	**Assignment**	**Frequency (cm^{-1})**	**Assignment**
3288.1	ν(OH) of water	3286.7	ν(OH) of water
2916.5	$\nu(CH_2)$ asymmetric	2916.1	$\nu(CH_2)$ asymmetric
2849.4	$\nu(CH_2)$ symmetric	2848.8	$\nu(CH_2)$ symmetric
1657.8	Amide I (α-helix)	1657.6	Amide I (α-helix)
1649.3	Amide I (random-coils)	1649.1	Amide I (random-coils)
1641.7	Amide I (random-coils)	1641.4	Amide I (random-coils)
1630.9	Amide I (β-sheet)	1630.9	Amide I (β-sheet)
1547.2	Amide II	1547.3	Amide II
1412.4	$\nu_{syn}(—COO^-)$	1410.5	$\nu_{syn}(—COO^-)$
1044.8	ν(C-OP) stretch	1047.9	ν(C-OP) stretch

S5 Elastic modulus and stress-strain response of reference elastomer

A bespoke micromanipulation system with a CCD camera was used for characterizing the mechanical properties of PDMS as a reference material for AFM nanoindentation. Details of the system configuration are described elsewhere [5]. Force transducers 403A and 405A (Aurora Scientific Inc., Canada) were mounted with end-polished 24 µm diameter glass needles. The system compliance was evaluated 5 times, and the mean value was used to compensate the force transducer and stage displacements. 10 randomly selected regions of a PDMS sample were indented until the maximum load was reached of about 7 mN, which corresponded to an indentation depth of ca. 60 µm. The resulting force displacement curves were found to be essentially linear up to indentation depths of ca. 20 µm and the fitted slopes used to calculate the elastic modulus using the theoretical relationship appropriate for a rigid flat punch indenting an isotropic linear elastic half-space [6]:

$$F = 2aE^*h \qquad \text{S22}$$

where the contact radius, *a*, is constant and equals the radius of the flat punch (12 µm). The mean plane strain elastic modulus, E^* = 3.79 ± 0.21 MPa, was obtained from the average slope of the 10 loading curves. The Young's modulus, *E*, and E^* are related by:

$$E^* = \frac{E}{1-\nu^2} \qquad \text{S23}$$

where ν is the Poisson's ratio of the PDMS, which was taken as 0.5 since it is considered incompressible. Hence *E* = 2.84 ± 0.16 MPa.

The tip calibration procedure requires that the PDMS behaves as a linear elastic half-space in the AFM nanoindentation experiments with a constant elastic modulus given by that obtained in microindentation using the flat punch. For this to be the case, it is necessary for the imposed elastic strain in both experiments to be within the linear elastic range. To calculate the representative elastic strain in these experiments, an expression is derived that is based on the force, F, as a function of indentation depth, h, for a power law indenter [7]:

$$F = E^* \frac{2n}{n+1}\left(\frac{1}{\kappa c}\right)^{1/n} h^{(n+1)/n} \qquad \text{S24}$$

where n, c, and κ are the tip geometry parameters, as described in S1. Hence, the mean contact stress can be described as a function of h and a as:

$$\sigma = \frac{F}{\pi a^2} = E^* \frac{2n}{n+1}\left(\frac{1}{\kappa c}\right)^{1/n} \frac{h^{(n+1)/n}}{\pi a^2} \qquad \text{S25}$$

The indentation depth can be expressed as a function of the contact radius, a:

$$h(a) = \kappa(n) c a^n \qquad \text{S26}$$

Combining Eqs S25 and S26 gives:

$$\sigma = \frac{E}{(1-\nu^2)} \frac{2n}{\pi(n+1)} \kappa c a^{n-1} = \varepsilon E \qquad \text{S27}$$

where ε is the representative elastic strain, which is therefore given by:

$$\varepsilon = \frac{1}{(1-\nu^2)} \frac{2n}{n+1} \frac{\kappa c a^{n-1}}{\pi} \qquad \text{S28}$$

or alternatively as:

$$\varepsilon = \frac{2n}{\pi(1-\nu^2)(n+1)} \frac{h}{a} \qquad \text{S29}$$

ε is generally termed a representative strain since the strain field under an indenter is not uniform and thus cannot be compared directly with, for example, that corresponding to simple uniaxial extension. In the limit, for indentation of PDMS (ν = 0.5) with a flat punch ($n \gg 1$) Eq. S29 reduces to:

$$\varepsilon = 0.85 \frac{h}{a} \qquad \text{S30}$$

For a 20 μm indentation depth with a = 12 μm, Eq. S30 gives ε = 1.4. Since the force-depth and stress-strain responses were found to be linear up to this strain, the elastic modulus is constant up to this value.

The average indentation depth applied to corneocytes in the AFM nanoindentation experiments was $h \approx 70$ nm. At this depth, for the AFM probe used, the strain was calculated using Eq. S29 to be ~ 1.16. Since this strain is within the linear elastic region observed in the microindentation experiments, this justifies the use of the elastic modulus value obtained for PDMS from the flat punch experiments in the AFM tip calibration procedure; at least for depths relevant to the corneocyte measurements. Fig. S6 shows a typical micromanipulator loading curve and its associated stress-strain curve (obtained using Eqs S25 and S30) plotted up to a strain of 1.16 ($h \approx 15$ μm). This illustrates the linearly elastic behaviour of PDMS in the range of strains relevant to the AFM corneocyte data analysis, which uses a tip shape function derived using the associated elastic modulus calculated from the average slope of the fitted curves.

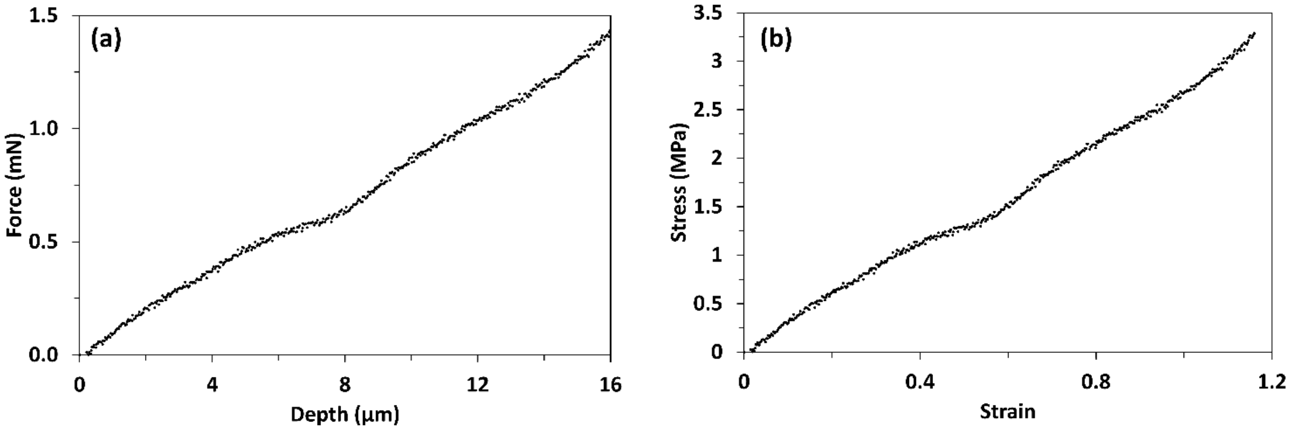

Fig. S6. (a) The force as a function of depth up to 15 μm for the indentation of PDMS with a flat punch using a micromanipulation technique. **(b)** The stress as a function of strain calculated from (a) using Eqs S25 and S30, respectively. The strain imposed at the maximum depth was 1.16.

S6 AFM nanoindentation of polymethyl methacrylate (PMMA)

A specimen of smooth cast PMMA sheet (Merck KGaA, Darmstadt, Germany) was used to demonstrate the utility and validity of the current tip calibration method. Nanoindentation experiments were performed using two different probes (cantilever A and B) and a range of maximum force setpoints for cantilever A (2.50, 3.40, 4.15 and 6.30 µN). The geometry of both probes was initially calibrated using the described methodology, i.e., by performing nanoindentation on the reference PDMS elastomer from which a tip contact radius function was obtained (Fig. S7). The measurements followed the described protocol, i.e., a total of 3 regions per cantilever and force setpoint with 64 loading and unloading force curves collected in a 5 x 5 µm region at a velocity of 0.5 µm/s. This included a dwell time of 4 s after loading to allow any viscous components to relax at the maximum force. The Oliver-Pharr method was employed to calculate the Young's moduli (assuming that ν = 0.35) from the unloading curves and the results are shown in Table S3. There is a relatively small variability between the data obtained from the 3 x 64 indents for each set point and close agreement between the five results. Moreover, the average values of the Young's moduli obtained (2.5–2.9 GPa) are satisfactorily close to the reported range for cast PMMA of 2.8–3.3 GPa [8].

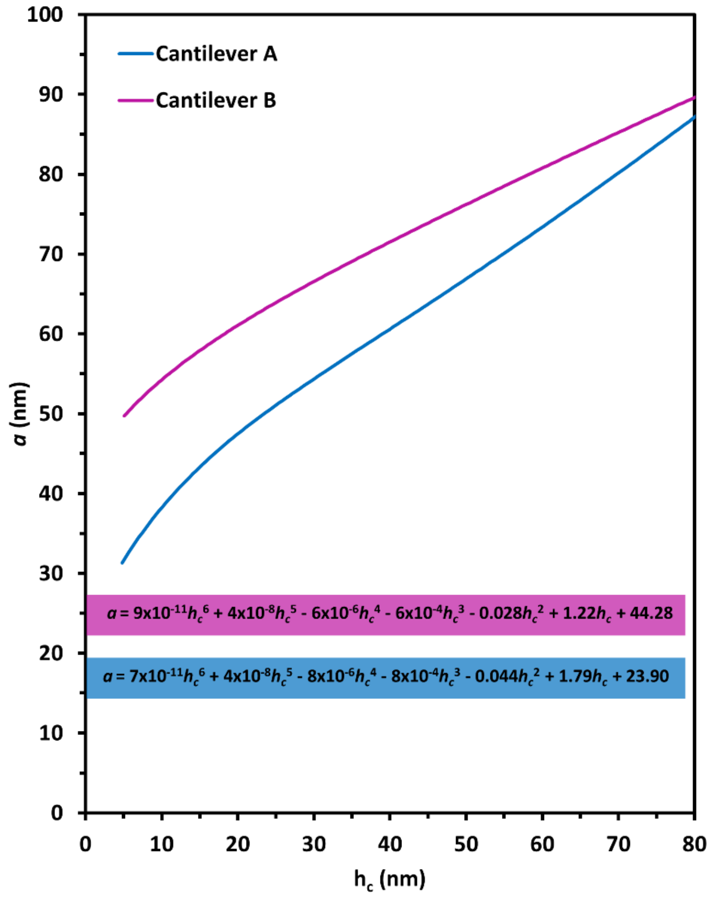

Fig. S7. The tip contact radius functions for two probes (Cantilever A and B) used for the nanoindentation of PMMA (average of three regions and 64 loading curves), which were obtained from a fit of $a(h_c)$ values from h_c = 5 to 150 nm.

Table S3. Young's moduli of PMMA obtained using two AFM probes and different maximum force setpoints.

Cantilever	Force (μN)	Indentation depth (nm)	Young's modulus (GPa)
A	**2.50**	18 ± 3	2.7 ± 0.4
	**3.40**	24 ± 4	2.6 ± 0.4
	**4.15**	32 ± 3	2.9 ± 0.4
	**6.30**	38 ± 3	2.5 ± 0.4
**B**	**3.40**	29 ± 10	2.7 ± 0.5

S7 Topography of the soft substrate - tape

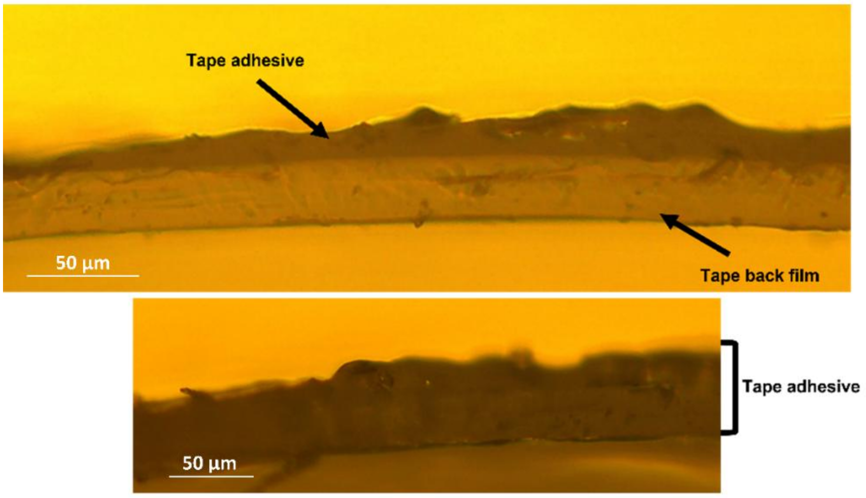

Fig. S8. Optical microscope images of Sellotape showing the two main parts of the tape: a backing film known as the carrier and a pressure-sensitive adhesive usually composed of polyacrylate derivatives. The adhesive layer is characterised by a certain roughness and variable thickness that will influence the interaction force with a cell.

S8 Lateral deflection of AFM probe tip

Huang et al. [9] have noted that, during indentation experiments, the flexure of a tilted cantilever will tend to generate an undesirable horizontal (lateral) displacement to the AFM probe tip. They proposed that the horizontal tip end deflection of the probe, Δx, can be calculated as follows [9]:

$$\Delta x = \Delta x_1 + \Delta x_2 \qquad \text{S31}$$

where Δx_1 is the horizontal translation of the tip base arising from the cantilever mounting angle, θ, and is given by:

$$\Delta x_1 = \Delta z \tan\theta \quad \text{S32}$$

where Δz is the vertical deflection of the probe tip arising from the flexure of the cantilever in response to the normal contact force. The vertical deflection is given by:

$$\Delta z = \frac{F}{k_c} \quad \text{S33}$$

where k_c is the AFM cantilever stiffness in the direction normal to the specimen surface.

Δx_2 is an additional component of the lateral deflection of the tip end caused by rotation of the tip base. This component is associated with the cantilever end deflection angle, $\Delta\theta$, created in response to the normal force. For a tilted cantilever beam with a force applied at the free end, this is given by [9]:

$$\Delta\theta = \frac{3\Delta z}{2L\cos\theta} \quad \text{S34}$$

where L is the AFM cantilever length. From simple geometry:

$$\Delta x_2 = \Delta\theta\, T\cos\theta \quad \text{S35}$$

where T is the AFM tip length. Hence:

$$\Delta x_2 = \frac{3T}{2L}\Delta z \quad \text{S36}$$

Therefore, from Eqs S31, S32 and S36, the ratio of the horizontal tip end deflection to the vertical deflection is given by [9]:

$$\frac{\Delta x}{\Delta z} = \left(\tan\theta + \frac{3T}{2L}\right) \quad \text{S37}$$

For the cantilevers used in the current experiments, T and L are given by the supplier as 15 μm and 117 μm, respectively, and $\theta = 10°$ (Nanowizard 4, Bruker, JPK BioAFM, Berlin, Germany). Hence, from Eq. S37, the ratio $\Delta x/\Delta z$ = 0.37 for the current cantilever geometry.

The stiffness of the AFM cantilever used for the PDMS indentation experiments was k_c = 28.5 N/m. Hence, at the maximum load of 250 nN (see S1D and Figure S1c), from Eqs S33 and S37, Δz = 8.8 nm and Δx = 3.2 nm respectively. The indentation depth at the maximum indentation load was typically h = 445 nm (S1D and Figure S1c). Hence, at the maximum load, the potential horizontal tip end deflection, Δx, is only 0.7% of the indentation depth, Δh. The shape of the loading curve is

such the $\Delta x/\Delta h$ ratio will be even less than this at smaller loads. Therefore, the effect of any lateral displacement on the AFM data for the elastomer was not significant.

For the case of corneocytes, similar calculations gave $\Delta x/\Delta h$ in the range 13–25% and, consequently, the lateral deflection is a significant proportion of the indentation depth. Nevertheless, this would be expected to have minor effects on the AFM corneocyte data since the cantilever end deflection angle, $\Delta\theta$, caused by the loads used in the current work is still < 0.03° (Eqs S33 and S34 with F_{max} = 1000 nN for corneocytes on glass, see Fig. 3b). The effects of the 10° mounting angle itself on the vertical component of the cantilever stiffness and the tip contact area are accounted for in the deflection sensitivity calibration procedure, and by using a reference sample of known elastic modulus.

However, Eqs S32 and S36 are derived assuming that there is not a resistance to the lateral tip displacement and that the tip end is free to rotate during contact. In practice, tangential contact forces associated with friction and lateral material deformation will reduce the lateral tip deflection. These tangential forces will also produce parasitic reductions in the cantilever end deflection angles that can result in significant underestimations of the normal force [9]. The phenomenon of lateral displacement errors in AFM nanoindentation could be addressed in future studies using strategies such as active lateral motion compensation [9], the use of non-deflecting cantilevers and reduced cantilever tilt angles [10-12].

S9 Cell thickness effect

The effect of the proximity of the underlying rigid support surface (in this case, a glass slide) on force curves was minimised by limiting maximum indentation depths to < 10% of the cell thickness. This is a criterion often used for thin film nanoindentation measurements of both elastic modulus and hardness [13]. Garcia et al. [14] examined the applicability of this criterion for conical and paraboloid AFM probe geometries, and derived the following generalised expressions for the respective indentation forces, F_{cone} and $F_{paraboloid}$:

$$F_{cone} = F_0\left[1 + \frac{0.721h\tan\alpha}{\delta} + \frac{0.65h^2\tan^2\alpha}{\delta^2} + \frac{0.491h^3\tan^3\alpha}{\delta^3} + \frac{0.225h^4\tan^4\alpha}{\delta^4}\right] \quad \text{S38}$$

where F_0 is the force for an infinite half-space, for which $F_{cone} = F_0$, α is the semi-included angle of the cone, and δ is the specimen thickness. For the indenter probe used in the current work, α ~ 22° (measured from an SEM image), δ was an average of 800 nm, and $h = h_{max}$ was limited to an average of 70 ± 20 nm to keep within the "10% rule" (h_{max}/δ = 9%). In this case, the sum of the second and higher order terms in Eq. S38 is 0.026, i.e., ca. 3% of F_0, which can be considered to be a small effect.

$$F_{paraboloid} = F_0\left[1 + \frac{1.133\sqrt{hR}}{\delta} + \frac{1.497hR}{\delta^2} + \frac{1.469hR\sqrt{hR}}{\delta^3} + \frac{0.755(h^2R^2)}{\delta^4}\right] \quad \text{S39}$$

where R is the end radius of the paraboloid. For the indenter probe used here, the approximate tip radius obtained from the tip check sample was R = 53 nm (see Fig. 1). Hence, with $h = h_{max}$ = 70 nm and δ = 800 nm, the sum of the second and higher order terms in Eq. S39 is 0.091, i.e., ca. 9% of F_0, which can be considered to also be a relatively small effect.

It's noted that the polynomials in Eq. S38 and Eq. S39 are essentially expressed in terms of the ratio of the contact radius to the specimen thickness, rather than simply the ratio of the indentation depth to the specimen thickness as the 10% rule would suggest.

Appendix: Nomenclature

Symbol	Description	Unit
a	contact radius	m
A_{max}	contact area at maximum indentation force of unloading curve	m^2
b	power law coefficient for loading curve	Nm^{-m}
b_i (i = 1, 2, 3, 4, 5, 6)	loading curve polynomial coefficients	Nm^{-i}
B_i (i = 0, 1, 2)	Prony series force relaxation coefficients	N
c	power law coefficient for indenter profile	m^{1-n}
C_e	power law coefficient for unloading curve	Nm^{-2}
C_i (i = 0, 1, 2)	Prony series hardness relaxation coefficients	Pa
d_i (i = 0, 1, 2, 3, 4, 5, 6)	indenter tip profile polynomial coefficients	m^{1-i}
E	Young's modulus	Pa
E^*	plane strain elastic modulus	Pa
F	indentation force	N
F_0	indentation force for an infinite half-space specimen	N
F_{cone}	indentation force for a conical tip (on a specimen of finite thickness)	N
F_{max}	maximum indentation force of unloading curve	N
$F_{paraboloid}$	indentation force for a parabolic tip (on a specimen of finite thickness)	N
h	indentation depth	m
h_c	contact depth	m
h_e	elastic depth recovery on unloading	m
h_f	residual indentation depth on unloading	m
h_{max}	indentation depth at maximum indentation force of unloading curve	m
h_s	surface elastic deflection at contact perimeter	m
H	hardness	Pa
H_0	instantaneous hardness	Pa
H_∞	long term hardness	Pa
i	summation index	1
j	plastic flow index	1
k	plastic flow consistency	$Pa.s^j$
k_c	AFM cantilever stiffness (in the vertical direction)	Nm^{-1}
L	AFM probe cantilever length	m
m	power law index for loading and unloading curves	1
n	power law index for indenter profile	1
R	radius of a spherical indenter, or end radius of curvature of a parabolic indenter	m
S	contact stiffness	Nm^{-1}

Symbol	Description	Unit
S_0	contact stiffness at maximum indentation force of unloading curve	Nm^{-1}
t	time	s
T	AFM probe tip length	m
Z	vertical AFM head height	m
α	semi-included angle of a conical indenter	rad
$\Gamma(.)$	gamma function	1
δ	specimen thickness	m
Δx	horizontal AFM probe tip deflection	m
Δx_1 , Δx_2	components of horizontal AFM probe tip deflection	m
$\Delta\theta$	AFM cantilever end deflection angle	rad
Δz	vertical AFM probe tip deflection	m
ε	strain	1
ε_e	elastic strain	1
ε_p	plastic strain	1
θ	AFM cantilever mounting angle	rad
κ	scaling factor (equals ratio of indentation depth to contact depth)	1
ν	Poisson's ratio	1
σ	unconfined bulk stress	Pa
$\bar{\sigma}$	mean contact stress	Pa
σ_Y	uniaxial yield stress	Pa
τ_1 , τ_2	Prony series relaxation times	s
ϕ	geometric factor for surface elastic deflection at contact perimeter	1
ψ	constraint factor for the mean contact stress	1

References

1. Oliver W. C., Pharr G. M. Measurement of hardness and elastic modulus by instrumented indentation: Advances in understanding and refinements to methodology. *J Mater Res* **19**, 3–20 (2004).
2. Garidel P. Mid-FTIR-Microspectroscopy of stratum corneum single cells and stratum corneum tissue. *Phys Chem Chem Phys* **4**, 5671–5677 (2002).
3. Lucassen G. W., van Veen G. N., Jansen J. A. Band analysis of hydrated human skin stratum corneum attenuated total reflectance fourier transform infrared spectra in vivo. *J Biomed Opt* **3**, 267–280 (1998).
4. Takada S., Naito S., Sonoda J., Miyauchi Y. Noninvasive in vivo measurement of natural moisturizing factor content in stratum corneum of human skin by attenuated total reflection infrared spectroscopy. *Appl Spectrosc* **66**, 26–32 (2012).
5. Zhang Z., Saunders R., Thomas C. R. Mechanical strength of single microcapsules determined by a novel micromanipulation technique. *J Microencapsul* **16**, 117–124 (1999).
6. Chen J. Nanobiomechanics of living cells: a review. *Interface Focus* **4**, 20130055 (2014).
7. Fischer-Cripps A. C. Contact mechanics. In: *Nanoindentation*. Springer New York (2002).
8. MatWeb. Overview of materials for Acrylic, Cast (www.matweb.com, 05/04/2024).
9. Huang L., Meyer C., Prater C. Eliminating lateral forces during AFM indentation. *J Phys: Conf Ser* **61**, 805–809 (2007).
10. Hoh J. H., Engel A., Friction effects on force measurements with an atomic force microscope, *Langmuir* **9**, 3310–3312 (1993).
11. Vanlandingham M. R., McKnight S. H., Palmese G. R., Eduljee R. F., Gillespie Jr J. W., McCulough R. L. Relating elastic modulus to indentation response using atomic force microscopy. *J Mater Sci Lett* **16**, 117–119 (1997).
12. Clifford C. A., Seah M. P., Quantification issues in the identification of nanoscale regions of homopolymers using modulus measurement via AFM nanoindentation, *Appl. Surf. Sci.* **252**, 1915–1933 (2005).
13. Zak S., Trost C. O. W., Kreiml P., Cordill M.J. Accurate measurement of thin film mechanical properties using nanoindentation. *J Mater Res* **37**, 1373–1389 (2022).
14. Garcia P. D., Garcia R. Determination of the elastic moduli of a single cell cultured on a rigid support by force microscopy. *Biophys J* **114**, 2923–2932 (2018).
