# Supplementary material for: Atomic force microscope protocols for characterising the elastoviscoplastic biomechanical properties of corneocytes
Source: Commun Biol. 2025 Dec 4;8:1747. doi: 10.1038/s42003-025-09142-0 (PMC12678835; doi:10.1038/s42003-025-09142-0)
Supplement: Supplementary file 3 — Description of Additional Supplementary Files [file 42003_2025_9142_MOESM3_ESM.docx]

1 Description of Additional Supplementary Files

2

1. **File name:** Supplementary Data 1
2. **Description:** Data file for all the calculations used in Figure 4 to 6, namely to calculate elastoviscoplastic properties of corneocytes and to derive power law fit presented in Figure 6.
3. **File name:** Supplementary Data 2
4. **Description:** Calculations performed to derive the geometry of the AFM tip using nanoindentation of PDMS and presented in Figure 2.
5. **File name:** Supplementary Data 3
6. **Description:** Data used in Figures 2, 3 and 5 of main manuscript.
7. **File name:** Supplementary Data 4
8. **Description:** Data used in Supplementary Figures.
9. **File name:** Supplementary Software 1
10. **Description:** Matlab scripts used for the analysis of all nanoindentation data obtained using AFM in this manuscript.

13

14

15
